# Iniencephaly: A Challenging Prenatal Diagnosis of a Neural Tube Defect

**DOI:** 10.7759/cureus.75457

**Published:** 2024-12-10

**Authors:** Lisandra Mendonça, Isabel Cerveira, Fernando Santos, Nuno Pereira, Joana Santos

**Affiliations:** 1 Gynecology and Obstetrics Department, Unidade Local de Saúde de Viseu Dão-Lafões, Viseu, PRT

**Keywords:** early prenatal ultrasound, iniencephaly, neural tube defects, prenatal diagnosis, rachischisis

## Abstract

Iniencephaly is a rare malformation of the base of the cranium, with an almost always fatal prognosis. This condition is part of the category of defects related to neural tube closure. Prenatal diagnosis can now be performed through ultrasound evaluation, allowing timely counseling. We present the case of a woman for whom an ultrasound diagnosis of iniencephaly at 13 weeks of gestation enabled early termination of the pregnancy.

## Introduction

Iniencephaly represents a neural tube defect involving alterations in the occiput bone and rachischisis of the cervical and thoracic spine with retroflexion of the head. It essentially manifests as an anomaly involving the craniovertebral junction, which is formed by articulations of the occipital condyles, the atlas, and axis vertebrae, causing an enlarged foramen magnum [1].

The etiology of this anomaly is multifactorial; nevertheless, factors such as syphilis, maternal ingestion of tetracyclines and sedatives, and consanguinity have been associated [2,3]. Additionally, Holmes et al. suggested that nutritional factors, including the mother's consumption of folic acid and multivitamins in the preconception period [1], may prevent the occurrence of iniencephaly.

The incidence of iniencephaly is estimated to be about 1/1,000-1/100,000 live births, depending on the geographical area, and it is more common in female sex [4]. Nowadays, the diagnosis is possible in the prenatal period through early ultrasound. Findings such as persistent dorsiflexion of the head, vertebral anomalies, and hydramnios are frequently seen [2,3]. Furthermore, anencephaly, encephalocele, meningomyelocele, hydrocephalus, Dandy-Walker malformation, holoprosencephaly, omphalocele, congenital diaphragmatic hernia, hydronephrosis, polycystic kidneys, cardiac defects, caudal regression sequence, arthrogryposis, club foot, single umbilical artery, and gastrointestinal atresia are among other abnormalities that may be linked to iniencephaly [5].

According to Lewis [6], iniencephaly can be classified into iniencephaly apertus when it is associated with encephalocele and iniencephaly clausus when no encephalocele is reported. Subsequently, another classification emerged, dividing iniencephaly into four groups: isolated iniencephaly, iniencephaly with encephalocele, iniencephaly with spina bifida, and iniencephaly with encephalocele and spina bifida [1,7].

The prognosis of the fetus with iniencephaly is reserved and almost always lethal. Many of the fetuses with iniencephaly are stillborn, although there have been reports in the literature of children and adults with iniencephaly with normal cognitive performance [7,8].

The main purpose of this article is to describe a case report of an early prenatal diagnosis of iniencephaly in Portugal, as no case reports of this anomaly have been reported in our country.

## Case presentation

A 25-year-old healthy Caucasian woman presented at our hospital for first-trimester screening. She had previously suffered from a spontaneous miscarriage at four weeks of gestation, with no intercurrences, and no family history of tubal neural defects or consanguinity was recounted. She had a body mass index (BMI) of 31 kg/m^2^ and had no alcohol or smoke addiction. The current pregnancy was spontaneously conceived, and she started multivitamins, including folic acid, one month before conception.

**Figure 1:**
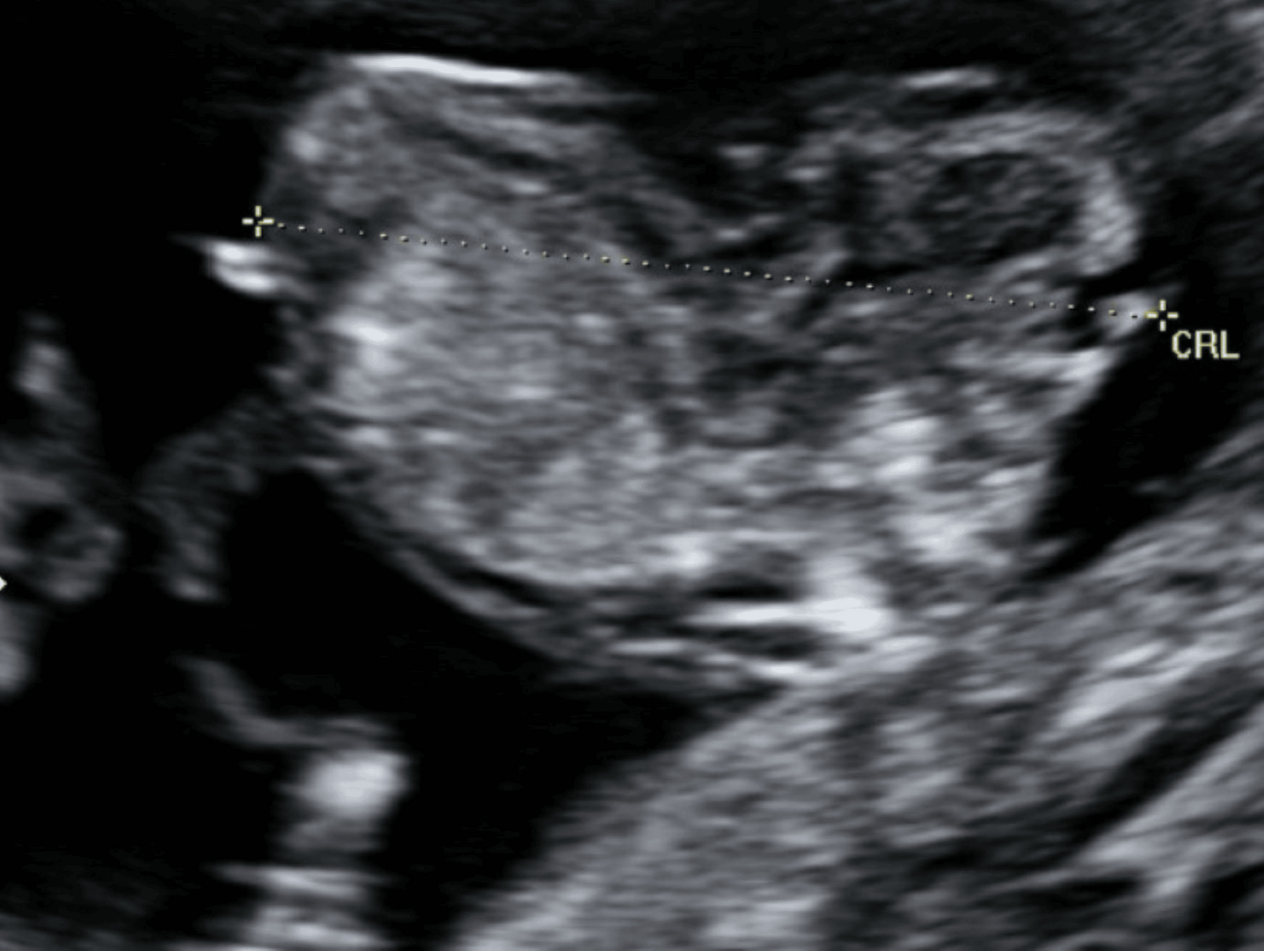
Ultrasound at 12 weeks and six days of gestation revealing the fetus with cervicothoracic hyperlordosis and absence of the neck

**Figure 2:**
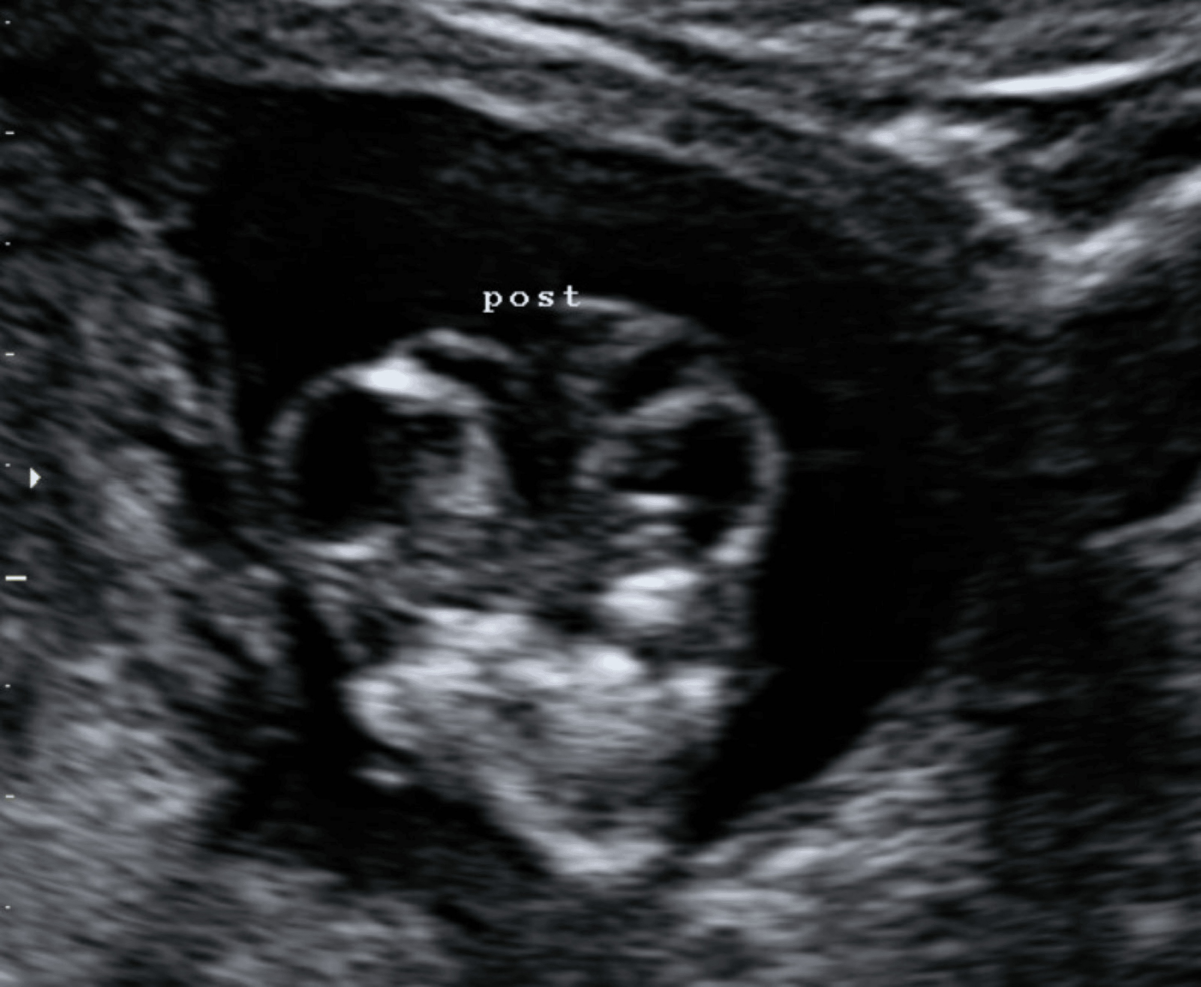
Transverse cervical section of the fetus in the ultrasound at 12 weeks and six days of gestation representing an open neural tube defect

Ultrasound at 12 weeks and six days of gestation revealed the presence of a fetus in a persistent hyperextension position and marked cephalic retroflexion. Additionally, the fetus presented a bulging of the abdominal wall as a consequence of a malformed cephalic pole, with an apparent open neural tube defect starting in the occipital region and extending to the thoracolumbar region (Figures 1, 2).

A chorionic villus sample was performed at 13 weeks and two days of gestation, and cytogenetic analysis revealed that it was a male fetus with a 46, XY karyotype without chromosomal abnormalities.

**Figure 3:**
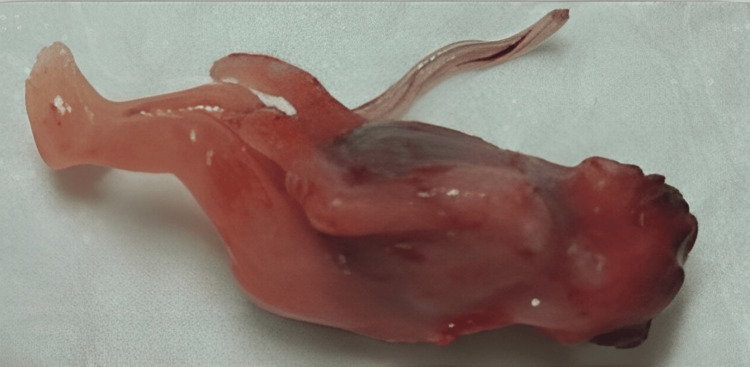
Autopsy front image of the fetus, showing a marked cephalic retroflexion and a bulging of the abdominal wall

**Figure 4:**
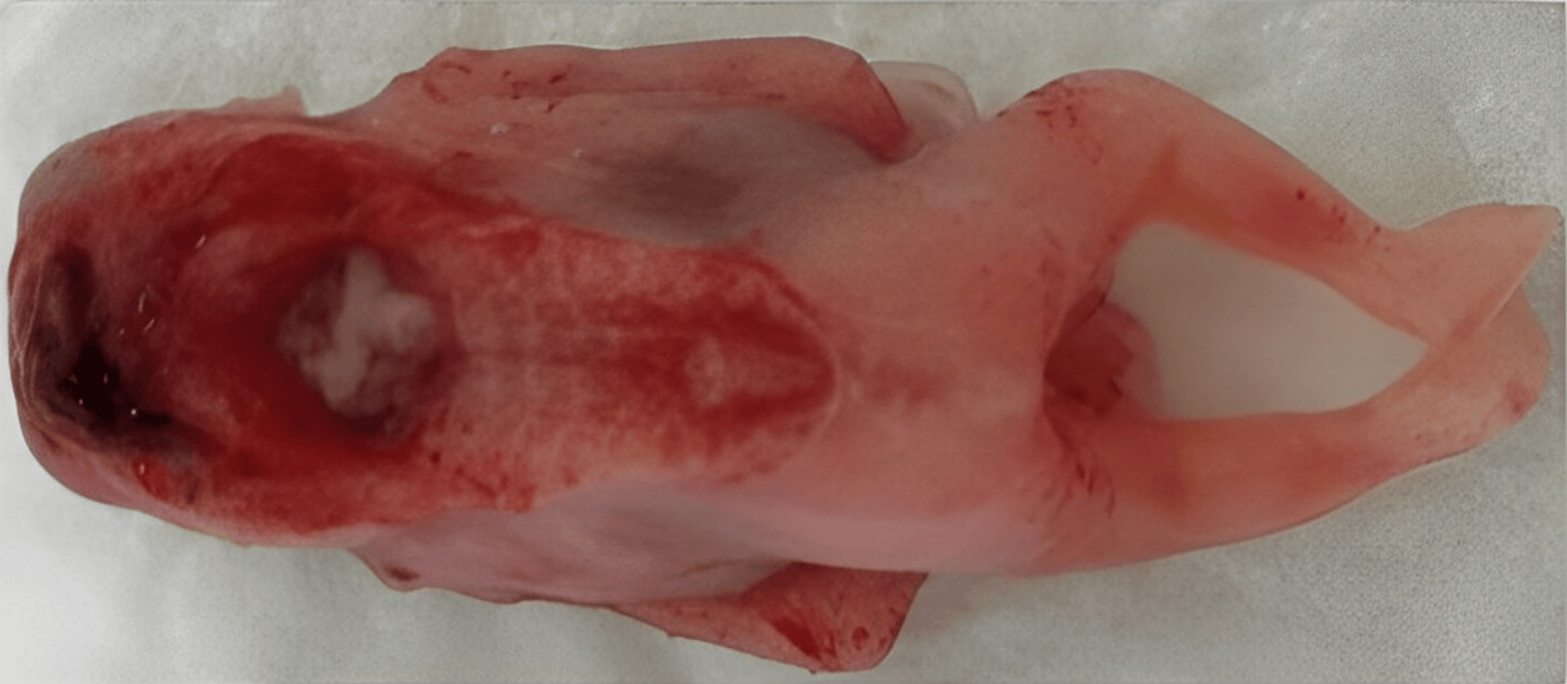
Autopsy posterior image of the fetus, revealing an open neural tube defect starting in the occipital region and extending to the thoracolumbar region

Based on these ultrasound findings and the poor prognosis, the couple opted for medical termination of the pregnancy (TOP), which was accepted by the hospital ethics committee. The TOP took place at 13 weeks and five days of gestation and was uneventful. The anatomopathological study of the fetus and placenta corroborated the ultrasound findings of iniencephaly (Figures 3, 4).

Those included microcephaly associated with the absence of bones in the cranial vault and a rudimentary occipital scale associated with exposure of the brainstem. The fetus, as expected, presented a short neck with hyperextension associated with extensive rachischisis and exposure of the spinal cord that ended at the level of the lumbar spine. Consequently, the fetus also presented bilateral ocular proptosis, a wide hypoplastic nose, prognathism, and low-set ears.

The autopsy showed several other associated malformations, including dental germ dysplasia, salivary hyperplasia, megaurethra, partial syndactyly of the second and third toes of the left foot, and dystrophic calcifications in the liver, and diffuse mineralization of intestinal contents. No signs of fetal infection were described.

Subsequently, the couple was counseled and advised to start a 5 mg folic acid supplementation three months before a new pregnancy. Furthermore, after six months, a new pregnancy occurred, and a healthy baby girl was born in the following year.

## Discussion

Neural tube defects are among the most common congenital anomalies. They occur when a part of the neural tube fails to close normally during the third and fourth weeks after conception, and they may involve the vertebrae, spinal cord, cranium, and/or brain [9]. Iniencephaly is considered a rare neural tube defect mainly characterized by a fixed retroflexion position of the cranium, a large occipital bone defect, and a posterior arch defect of the cervical spine.

The etiopathogenesis of iniencephaly is unknown, and it is unclear whether it is caused by a closure failure of the neural tube or due to the dehiscence of a previously closed neural tube, although the last theory is the most accepted. Moreover, iniencephaly is commonly attributed to genetic causes such as monosomy X, trisomy 13, trisomy 18, and other chromosomal abnormalities [10]. For this reason, we conducted a chromosomal analysis that was normal for the male sex, even though the current literature indicates a higher prevalence of iniencephaly in the female sex, in a proportion of 9:1 [11,12]. A family history of neural tube defects has been linked with a recurrence rate of iniencephaly of 5%, which is why proper counseling is important before conception [10].

Other factors have been implicated as possible etiologic factors, such as certain drugs, maternal syphilis, poor socioeconomic conditions, lack of folic acid supplementation, and, as in our case, obesity. Complex anomalies, oligohydramnios, and maternal obesity may challenge early prenatal ultrasound diagnosis [10]. In our case, maternal BMI was equivalent to class I obesity, although it did not appear to affect the correct diagnosis. A differential diagnosis of prenatal diagnosis of iniencephaly should include conditions involving cervical hyperextension and nuchal tumors, namely teratoma, goiter, lymphangioma, cervical myelomeningocele, encephalocele, Klippel-Feil syndrome, and Jarcho-Levin syndrome [12].

Khatri et al. [10] demonstrated that nearly 250 cases of this anomaly have been reported in the literature, and only eight patients have survived long-term. The high mortality rate of iniencephaly is believed to be related to associated malformations apart from the central nervous system. Cardiovascular, urinary, skeletal, and gastrointestinal systems are commonly affected by iniencephaly and malformations such as facial dysmorphism, cleft lip and palate, congenital diaphragmatic hernia, intestinal atresia, imperforate anus, horseshoe kidney, bronchogenic cysts, and pulmonary hypoplasia should be sought [10].

This case report highlights the importance of early prenatal ultrasound in making an accurate diagnosis of iniencephaly. In particular, appropriate and timely prenatal diagnosis enables parental counseling and prompt TOP if intended. 

One important limitation of this case report is the absence of a documented body X-ray.

## Conclusions

Neural tube defects have a multifactorial etiology. Iniencephaly is considered a rare manifestation of a neural tube defect with a very poor prognosis. In this context, a detailed early ultrasound evaluation, including neurosonography, is essential to promote timely counseling for the couple in current and future pregnancies. Additionally, prompt folic acid supplementation at least three months before conception is recommended.

The associated anomalies described in this case are closely linked with neural tube defects, meaning there is no significantly increased risk of independent recurrence. A well-documented fetopathological examination is mandatory to confirm the diagnosis and categorize associated malformations to better understand the pathophysiology of iniencephaly.
